# Bilateral Axillo-Brachial Artery Stenosis Following Messenger Ribonucleic Acid (mRNA) Vaccination Against Severe Acute Respiratory Coronavirus 2 (COVID-19)

**DOI:** 10.7759/cureus.33843

**Published:** 2023-01-16

**Authors:** Hasan Güven

**Affiliations:** 1 Interventional Cardiology, Grandview Medical Center, Birmingham, USA

**Keywords:** covid-19 mrna vaccine, endovascular angioplasty, vasculitis, giant cell arteritis, brachial artery occlusion

## Abstract

The following case report is an overview of an unusual presentation of bilateral axillo-brachial artery occlusion following messenger ribonucleic acid (mRNA) vaccination against severe acute respiratory coronavirus 2 (COVID-19). A 64-year-old female presented with symptoms initially consistent with polymyalgia rheumatica five weeks following the first booster of the Pfizer-BioNTech COVID-19 vaccine. She was successfully treated with prednisone therapy; however, despite the normalization of inflammatory markers, she later presented with bilaterally occluded axillo-brachial arteries. She successfully underwent endovascular management for the treatment of her symptoms. To our knowledge, this is the first case report of chronically occluded bilateral axillo-brachial artery disease following mRNA vaccination for COVID-19 successfully treated with endovascular therapy. The unusual pathogenesis of upper extremity arterial disease is reviewed and a review of endovascular treatment options is presented. A literature review of the types of vasculitis seen following mRNA COVID-19 vaccination is also presented.

## Introduction

The following case is an overview of an unusual presentation of bilateral axillo-brachial artery occlusion following the messenger ribonucleic acid (mRNA) vaccine against severe acute respiratory coronavirus 2 (COVID-19). Upper extremity occlusive vascular disease and, in particular, axillo-brachial artery stenosis is uncommonly encountered in patients [1]. It has been described in patients who have undergone upper extremity traumatic injuries as well as cases of vasculitis [2]. To our knowledge, the endovascular treatment of bilateral symptomatic axillo-brachial artery occlusion following COVID-19 mRNA vaccination has not been described in the literature. This case report hopes to review the presentation and treatment options available for this uncommon disorder. Furthermore, a literature review is provided to investigate the association of this issue following COVID-19 mRNA vaccination.

## Case presentation

The patient is a 64-year-old female with an unremarkable past medical history who began to develop upper extremity weakness and bilateral bicep muscle tenderness occurring five weeks following the first booster with the Pfizer-BioNTech (Pfizer, New York, USA, and BioNTech, Mainz, Germany) mRNA vaccine against COVID-19. She initially noticed right arm weakness while driving her vehicle which progressed to involve the left arm within one week. She subsequently developed fatigue, anorexia, hair loss, weight loss, and weakness. She had some jaw discomfort with mastication. She also complained of coolness on the dorsum of her hands. She denied any fever. Neurologic evaluation including the brain and cervical MRI along with nerve conduction studies was unremarkable. She was subsequently seen by the Rheumatology team and was noted to have a significantly elevated sedimentation rate (50 mm/hour) as well as C-reactive protein levels (104 mg/L). Creatinine kinase levels were noted to be normal. Temporal artery ultrasound imaging showed no suggestion of temporal artery arteritis. On examination, she was noted to have bilateral weak pulses in her upper extremities with an inability to record blood pressure with an automatic cuff. She underwent initial evaluation with upper extremity arterial ultrasound studies which showed diminished flow velocities involving bilateral brachial, radial, and ulnar arteries without focal stenosis appreciated although the monophonic waveforms seen on the ultrasound were highly suggestive of proximal high-grade stenosis. In addition, some of the views of her left axillary artery were notable for abnormal intimal media thickening (possible “halo” sign). Her presentation was felt to be most consistent with polymyalgia rheumatica (PMR) with possible arteritis. She was placed on prednisone 20 mg daily with significant improvement of symptoms within one week with normalization of inflammatory markers. She was weaned off of steroids seven months following her presentation with continued normalization of inflammatory markers.

**Figure 1:**
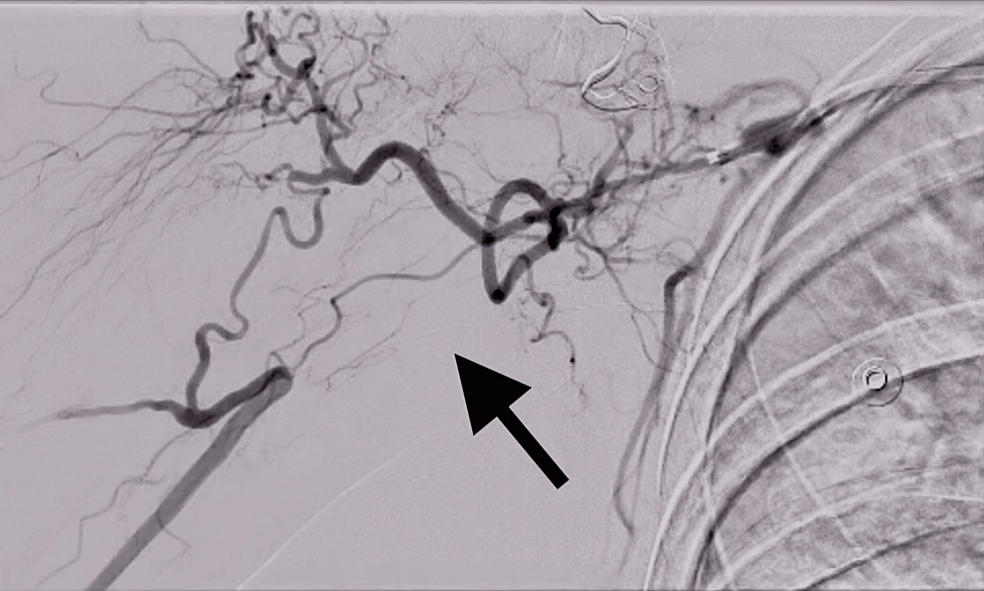
Angiogram of the right upper extremity demonstrating right axillo-brachial artery chronic occlusion (denoted by black arrow).

**Figure 2:**
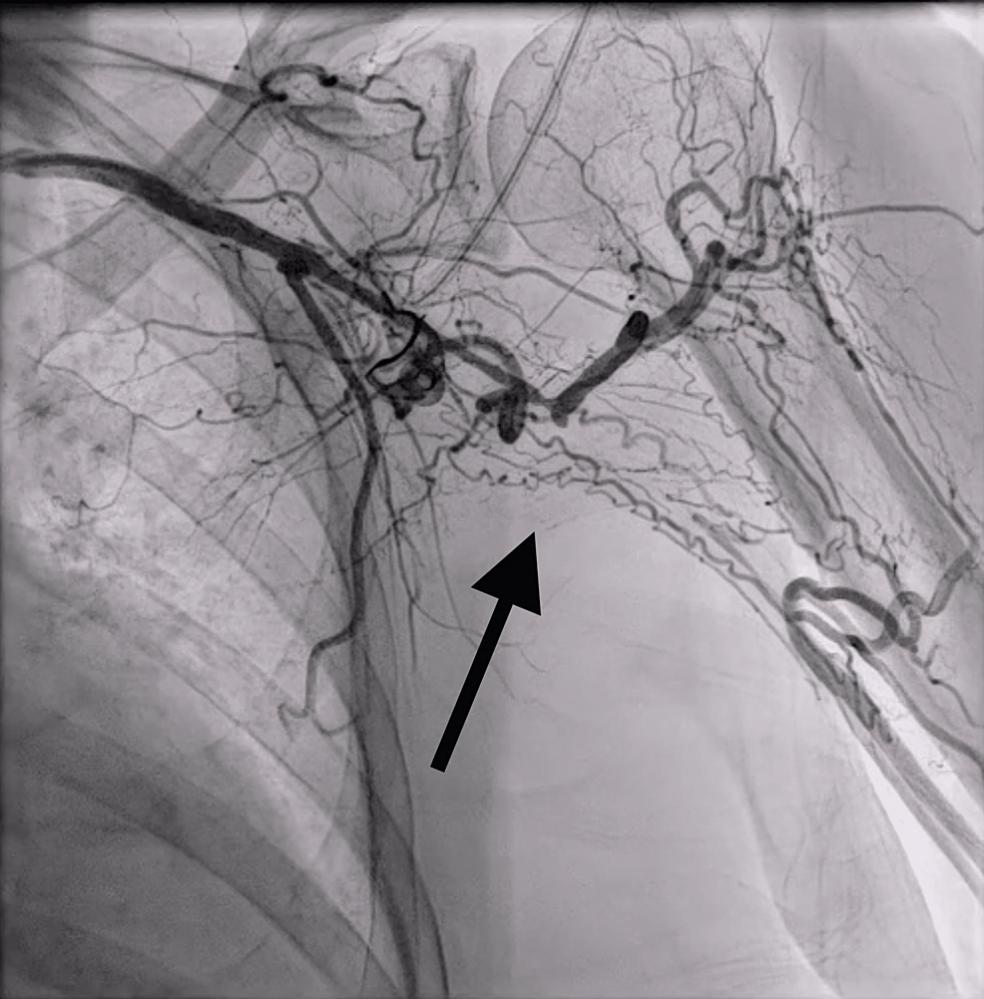
Angiogram of the left upper extremity demonstrating left axillo-brachial artery chronic occlusion (denoted by black arrow).

Given persistent bilateral weak pulses and the inability to measure automated brachial blood pressures, she was referred to cardiology. The examination was notable for 1+ radial, ulnar, and brachial pulses. She underwent a CT angiography of the thoracic aorta and great vessels which was unremarkable. Distal subclavian and axillary arteries were not well seen. Given persistent coolness to the dorsum of the hands and some hand tingling, the patient elected to undergo an invasive evaluation of upper extremity vasculature. Angiogram was performed which revealed occlusion of bilateral axillary and brachial arteries with collateralization (Figures 1, 2).

She was monitored clinically; however, given persistent symptoms, her case was discussed with the vascular team and an initial endovascular revascularization approach was recommended.

**Figure 3:**
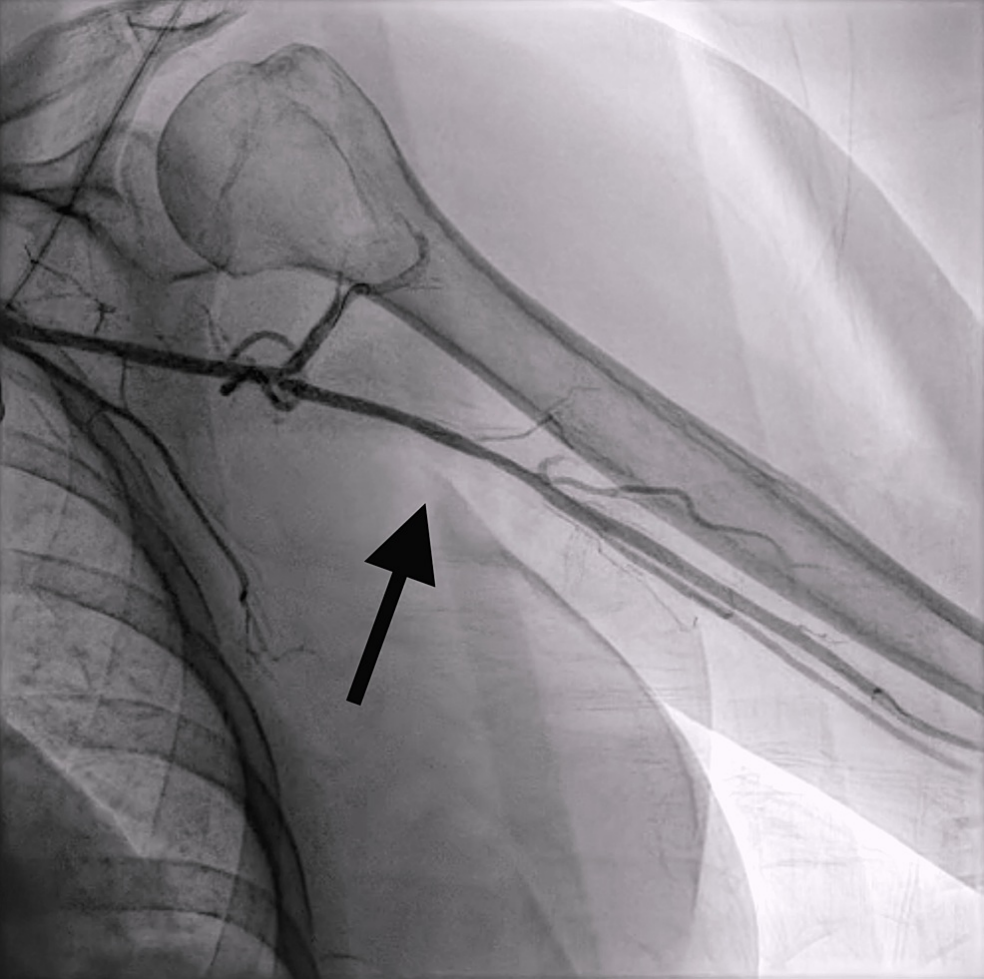
Left upper extremity angiogram demonstrating ravascularization of the left axillo-brachial artery following endovascular therapy (denoted by black arrow).

Initially, revascularization of the left brachial artery was planned. Access was obtained from the left radial artery as well as the right common femoral artery utilizing ultrasound guidance. Heparin was used as an anti-thrombotic agent. The left subclavian artery was engaged with a 6-French Judkins right 4 guide catheter (Medtronic Inc, Minneapolis, USA) and brought in proximity to the occlusion with the aide of a 0.035-inch wire. Subsequently, the proximal occlusion was traversed from a retrograde approach with a Trailblazer catheter (Medtronic Inc, Minneapolis, USA) in conjunction with a knuckled Terumo glidewire (Terumo Interventional Systems, Tokyo, Japan). Subsequently, a Mongo 0.014 inch Asahi wire (Asahi Intecc USA Inc, Irvine, USA) was utilized to complete the crossing of the lesion; however, the Trailblazer could not be traversed more proximally. At this point, a balloon angioplasty was performed with Trek 1.2 × 12 mm as well as 2.5 × 30 mm balloons (Abbott Vascular, Santa Clara, USA). Subsequently, the lesion was easily wired in an antegrade fashion. A 4 × 100 mm AngiogSculpt balloon (Philips Healthcare, Best, Netherlands) was used for angioplasty which led to excellent flow into the arm with no hemodynamic gradient seen across the stenosis (Figure 3) (post-angioplasty result).

**Figure 4:**
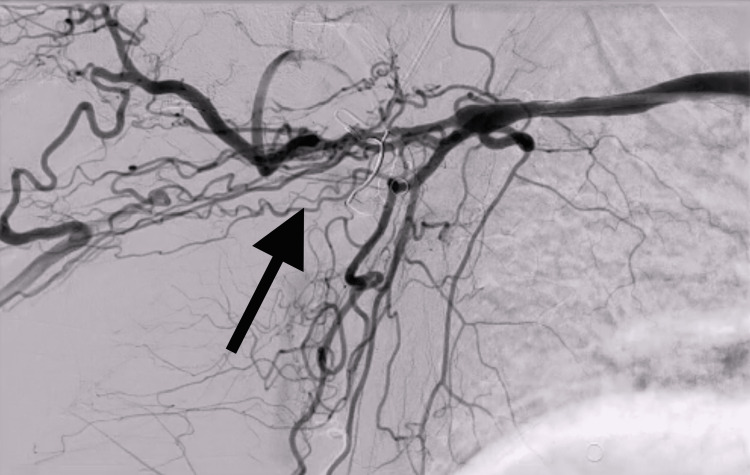
Digital subtraction angiography of the right upper extremity demonstrating the ambiguous proximal cap of the right axillo-brachial artery with extensive collateralization (denoted by black arrow).

Given the significant improvement in symptoms in the left arm, the patient elected to undergo endovascular revascularization of the right arm. Right femoral and right radial access was obtained with ultrasound guidance, and heparin was again utilized as the anti-thrombotic agent. The distal cap could be traversed in a retrograde fashion with a Trailblazer in conjunction with a Terumo Runthrough as well as Asahi Mongo 0.014-inch wires; however, the proximal cap could not be crossed. With antegrade imaging, the proximal cap was difficult to discern (Figure 4).

**Figure 5:**
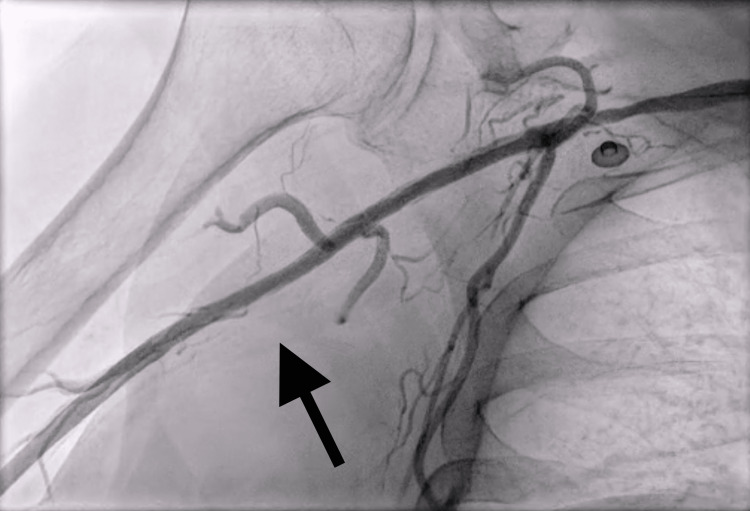
Angiogram of the right upper extremity demonstrating revascularization of the right axillo-brachial artery following endovascular therapy (denoted by black arrow).

Utilizing an Asahi Pilot 50 0.014-inch wire the proximal cap was crossed which subsequently allowed a Trailblazer to cross into the true lumen of the brachial artery distally. Once true lumen location was confirmed angiographically, the artery was dilated with a Medtronic Nanocross 2.0 × 60 mm followed by a 4 × 120 mm balloon. To optimize luminal gain, several prolonged inflations up to 180 seconds were performed with a Cordis Chocolate 5 × 120 mm balloon (Cordis Corporation Santa Clara, USA). Post-angioplasty, there were good angiographic results without significant gradient across the diseased segment (Figure 5). At the three-month follow-up, the patient was noted to have a resolution of symptoms.

## Discussion

Axillo-brachial artery stenosis (distal upper extremity stenosis) is an uncommon presentation in patients with upper extremity arterial disease, with innominate and subclavian stenosis being more common entities largely attributable to atherosclerosis [1]. The disease process has been described following trauma to the upper extremity including patients who acquired the disease after utilizing crutches for ambulatory support [3]. Brachial artery stenosis has been described in baseball pitchers and has been felt to be due to overuse and recurrent injury to the vessel [4]. In addition, radiation-induced upper extremity arterial stenosis can be seen [5]. Upper extremity vascular disease can be seen in the setting of vasculitis and has been described in the setting of giant cell arteritis as well as Takayasu’s arteritis, although distal involvement is more commonly seen with giant cell arteritis (GCA) [6]. Stenosis of the brachial artery due to fibromuscular dysplasia has also been seen [7]. Sporadic cases of unclear etiology have been described in the literature [8].

Numerous modalities have been utilized for the treatment of brachial artery stenosis. Treatments ranging from standard balloon techniques in addition to stenting with bare metal stents, angioplasty with drug-eluting balloons, atherectomy as well as surgical revascularization have been reported in the literature [8-12]. No definitive standard guidelines are currently available to guide the treatment of axillo-brachial artery stenosis. However; the long-term patency of endovascular therapy is noted to be favorable in the case reports cited.

The association of vasculitis with COVID-19 mRNA vaccination has been described. Case reports have included cases of immunoglobulin A vasculitis, lymphocytic vasculitis, anti-neutrophilic cytoplasmic autoantibody-associated vasculitis, leukocytoclastic vasculitis, urticarial vasculitis, and immune complex vasculitis [13-15].

In early 2022, several case reports of large vessel and GCA developing after mRNA COVID-19 vaccination were published with multiple authors demonstrating the utility of fluorodeoxyglucose-positron emission tomography in conjunction with computed tomography imaging for diagnosis [16-24].

Interestingly, some centers have reported an increased incidence of GCA during the COVID-19 pandemic [25]. Furthermore, there are case reports of GCA being triggered by COVID-19 infection as well [26].

There has been some concern that mRNA vaccination can disrupt endothelial function, with a study demonstrating decreased brachial artery flow-mediated vasodilatation following the second dose of the mRNA vaccine [27].

It is, however, encouraging that a global pharmacovigilance study to investigate the risk of GCA and PMR following mRNA vaccination for COVID-19 did not demonstrate an increased reporting of these diseases when compared to reporting for side effects following vaccination for influenza in a World Health Organization global individual case safety report database [28].

This case report illustrates that techniques developed for the endovascular treatment for chronic occlusion of lower extremity vasculature can also be utilized to revascularize chronic occlusion of upper extremity vessels. These endovascular options allow patients an effective treatment strategy for improved quality of life without significant morbidity. A review of the literature suggests that endovascular treatment options provide favorable long-term outcomes for patients. Emerging case reports suggest that there is a possibility of an association between the mRNA vaccine against COVID-19 and large vessel vasculitis. Case reports need to be followed to determine whether these associations are merely seen by chance in patients who were likely to show a phenotype of vasculitis regardless of being vaccinated against SARS-CoV-2. The possibility that vaccination could be potentiating an underlying mechanism for large vessel vasculitis in patients with an underlying susceptibility to this disease needs to be investigated.

## Conclusions

The possibility of upper extremity occlusive disease should be considered in patients who present with unusual upper extremity symptoms following COVID-19 or vaccination against COVID-19. Endovascular options allowed this patient to undergo revascularization without significant morbidity. If restenosis develops in this patient, further endovascular options include angioplasty with drug-eluting balloons versus utilization of stenting or surgery. In the short term, the patient remains free of vascular complaints.
